# Enhancement of Lipid Production under Heterotrophic Conditions by Overexpression of an Endogenous bZIP Transcription Factor in *Chlorella* sp. HS2

**DOI:** 10.4014/jmb.2005.05048

**Published:** 2020-07-29

**Authors:** Hansol Lee, Won-Sub Shin, Young Uk Kim, Seungjib Jeon, Minsik Kim, Nam Kyu Kang, Yong Keun Chang

**Affiliations:** 1 Department of Chemical and Biomolecular Engineering, Korea Advanced Institute of Science and Technology (KAIST), Daejeon 344, Republic of Korea; 2 Advanced Biomass R&D Center, Daejeon 34141, Republic of Korea; 3 Human Convergence Technology Group, Korea Institute of Industrial Technology (KITECH), Ansan 15629, Republic of Korea; 4 Carl R. Woese Institute for Genomic Biology, University of Illinois at Urbana-Champaign, Urbana, IL 61801, USA

**Keywords:** Microalgae, *Chlorella* sp. HS2, bZIP transcription factor, fatty acids, biofuels, heterotrophic cultivation

## Abstract

Transcription factor engineering to regulate multiple genes has shown promise in the field of microalgae genetic engineering. Here, we report the first use of transcription factor engineering in *Chlorella* sp. HS2, thought to have potential for producing biofuels and bioproducts. We identified seven endogenous bZIP transcription factors in *Chlorella* sp. HS2 and named them HSbZIP1 through HSbZIP7. We overexpressed HSbZIP1, a C-type bZIP transcription factor, in *Chlorella* sp. HS2 with the goal of enhancing lipid production. Phenotype screening under heterotrophic conditions showed that all transformants exhibited increased fatty acid production. In particular, HSbZIP1 37 and 58 showed fatty acid methyl ester (FAME) yields of 859 and 1,052 mg/l, respectively, at day 10 of growth under heterotrophic conditions, and these yields were 74% and 113% higher, respectively, than that of WT. To elucidate the mechanism underlying the improved phenotypes, we identified candidate HSbZIP1-regulated genes via transcription factor binding site analysis. We then selected three genes involved in fatty acid synthesis and investigated mRNA expression levels of the genes by qRTPCR. The result revealed that the possible HSbZIP1-regulated genes involved in fatty acid synthesis were upregulated in the HSbZIP1 transformants. Taken together, our results demonstrate that HSbZIP1 can be utilized to improve lipid production in *Chlorella* sp. HS2 under heterotrophic conditions.

## Introduction

Increasing concerns about fossil fuels, including their limited supply and contribution to climate change, have triggered a growing interest in sustainable energy. Various sustainable sources of energy have arisen as alternatives to fossil fuels; among the possible sources, microalgae are considered the most outstanding feedstock [1]. When compared to first-generation (corn and soybeans) and second-generation (lignocellulosic feedstock) biomasses, microalgae do not have a large-scale requirement for agricultural land. Microalgal cultivation takes a relatively short time, resulting in a high biomass yield. Microalgae also do not create the ethical problem of using food products, which is an important limitation of first-generation biomasses. Furthermore, microalgae can be used in various ways, such as in health foods, cosmetics, biofuels, and industrial materials [2-4]. However, despite the advantages of microalgae, the commercialization of microalgal biofuels and biomaterials has been limited by their high production cost [5]. The bioprocessing of microalgae requires cultivation, harvesting, extraction, and conversion. For the purpose of industrial production, each step needs to be optimized to make the overall process economically feasible [6].

A cost-effective microalgal bioprocess could be designed using strains developed through genetic engineering. Among the strategies for genetic engineering of microalgae, transcription factor engineering (TFE) has gained attention [7, 8]. Since microalgae possess various organelles and complicated metabolic pathways, it is difficult to reliably achieve desirable phenotypes by regulating individual metabolic enzymes. In contrast, as TFE can alter expression levels of multiple metabolic genes simultaneously, it is considered an effective method of genetic manipulation in microalgae. Indeed, the overexpression of the endogenous bHLH and bZIP TFs in *Nannochloropsis salina* positively affected lipid production by regulating transcript levels of lipid synthesis genes [9-11]. It has also been reported that the heterologous expression of plant TFs in microalgae increased lipid production by introducing novel regulatory systems. [12-14].

Heterotrophic cultivation can be another effective way to industrially produce microalgae-based bioproducts [15]. Unlike photoautotrophic cultivation, where microalgae apply photosynthesis to utilize inorganic carbon in the form of CO_2_, microalgae under heterotrophic cultivation use organic carbon sources without photosynthesis [16]. The latter process does not require CO_2_ concentration, CO_2_ fixation or gluconeogenesis, and is thus much more efficient than the metabolism that takes place in photoautotrophic culture [17, 18]. Consequently, biomass and lipid productivities under heterotrophic culture conditions can be enhanced compared to photoautotrophic culture. In addition, since light illumination is not necessary for heterotrophic cultivation, any conventional fermenter designed for industrial production can be used for heterotrophic cultivation of microalgae [19]. Furthermore, life-cycle assessments performed in the context of biodiesel production have shown that heterotrophic cultivation could reduce the global warming potential and improve the net energy ratio for algal biodiesel compared to phototrophic cultivation [20, 21].

In this study, we demonstrate the first application of TFE to *Chlorella* sp. HS2, which has been spotlighted as an industrial strain [22]. We identified the endogenous bZIP TFs in *Chlorella* sp. HS2 and overexpressed HSbZIP1, a C-type bZIP TF (identified by comparison to the *Arabidopsis* bZIP TFs) that has high homology to NsbZIP1, which was previously shown to enhance lipid production in *Nannochloropsis salina* [10]. HSbZIP1-overexpressing transformants grown under the heterotrophic condition exhibited increased lipid production. We identified possible HSbZIP1-regulated genes involved in lipid production and determined their expression levels by quantitative real-time PCR (qRT-PCR). Our observation that lipid production under heterotrophic cultivation can be increased by overexpression of HSbZIP1 suggests that this strategy could be used for industrial biofuel production in microalgae.

## Materials and Methods

### Microalgal Strains and Culture Conditions

*Chlorella* sp. HS2 was obtained from the Korea Research Institute of Bioscience and Biotechnology (KRIBB), Republic of Korea [22]. This strain was maintained in Tris-Acetate-Phosphate medium (TAP medium), which consisted of Trizma base (2.42 g/l), NH_4_Cl (375 mg/l), MgSO_4_·7H_2_O (100 mg/l), CaCl_2_·2H_2_O (50 mg/l), K_2_HPO_4_ (108 mg/l), KH_2_PO_4_ (54 mg/l), 1 ml/l of glacial acetic acid and 1 ml/l of Hutner’s trace metal solution (50 mg/l Na_2_EDTA·2H_2_O, 22 mg/l ZnSO_4_·7H_2_O, 11.4 mg/l H_3_BO_3_, 5.06 mg/l MnCl_2_·4H_2_O, 4.99 mg/l FeSO_4_·7H_2_O, 1.61 mg/l C°Cl_2_·6H_2_O, 1.57 mg/l CuSO_4_·5H_2_O and 1.1 mg/l (NH_4_)_6_Mo_7_O_24_·4H_2_O). The cells were grown at 32°C under fluorescent light (120 μmol photons/m2/s) with shaking at 120 rpm.

### Vector Construction

The HSbZIP1 gene was cloned into a pUC19 plasmid [23] to generate pHSbZIP1. The coding sequence (CDS) of HSbZIP1 was amplified from *Chlorella* sp. HS2 cDNA. Total RNA was isolated using an RNeasy Plant Mini Kit (Qiagen, USA) and the residual DNA was removed using a DNA-free DNase Kit (Ambion, USA) according to the manufacturer’s instructions. Superscript III Reverse Transcriptase (Invitrogen, USA) and an oligo (dT)_20_ primer (Invitrogen) were used to synthesize cDNA. The HSbZIP1 CDS was amplified with primers HSbZIP1-fwd and HSbZIP1-rev (Table S1). The backbone of pHSbZIP1 was amplified with primers pHSbZIP1-fwd and pHSbZIP1-rev (Table S1). *HsbZIP1* and the pHSbZIP1 backbone were combined by the Gibson assembly technique [24]. The HSbZIP1 harbored in the pHSbZIP1 plasmid was expressed using the 29B HSP70 promoter and the Nos terminator, and the *Aph7* gene, which confers resistance to hygromycin B, was expressed using the b2 TUB promoter and the Rbc S2 terminator.

### Transformation of *Chlorella* sp. HS2

Electroporation was used for the transformation of *Chlorella* sp. HS2. *Chlorella* sp. HS2 cells were grown in TAP medium at 32°C with agitation at 120 rpm under 120 μmol photons/m2/s of fluorescent light. The cells were harvested at the exponential phase, and 4 × 107 cells were concentrated and suspended with 80 μl of MAX Efficiency Transformation Reagent for Algae (ThermoFisher Scientific, USA). Thereafter, 5 μg of pHSbZIP1 vector linearized by SspI was added to the cell-buffer mixture. The DNA and cell-buffer mixture were transferred to a 2-mm gap electroporation cuvette (BTX, USA) and kept on ice for 10 min. A Bio-Rad Gene Pulser Xcell electroporation system (Bio-Rad, USA) was used at 1,500 V/cm, 200 Ω and 25 μF with a single exponential decay pulse. After electroporation, the cells were kept on ice for 10 min and then transferred to a Falcon tube (Fisher Scientific) containing 3 ml of TAP medium with 40 mM sucrose and 100 μg/ml ampicillin. The cells were recovered in the dark at 27°C for 24 h, harvested by centrifugation and then suspended in 300 μl of TAP medium with 40 mM sucrose. The suspended cells were plated onto TAP agar plates containing 500 μg/ml hygromycin and incubated at 25°C under continuous fluorescent light of 120 μmol photons/m^2^/s.

### Molecular Analysis of the *HsbZIP1* Transformants by Genomic DNA PCR and Western Blotting

Transfer of the pHSbZIP1 vector was confirmed by genomic DNA PCR. The genomic DNA was extracted by Instagene Matrix (Bio-Rad) following the manufacturer’s instructions [25]. The transgene was detected using primers CPC1-fwd and CPC1-rev, and the 18S ribosomal DNA was detected as a loading control using primers 18S-1-fwd and 18S-1-rev (Table S1). Ex-Taq polymerase (Takara, Japan) was used for PCR amplification under the following conditions: 95°C for 5 min; 30 cycles of 95°C for 1 min, 60°C for 1 min and 72°C for 1 min; and then 72°C for 10 min. The expected sizes of the PCR products for the transgene and 18S rDNA were 754 and 139 bp, respectively.

For western blotting, 10^8^ cells in mid-exponential phase were harvested, re-suspended in 200 μl of deionized water, and mixed with 100 μl of Laemmli sample buffer cocktail containing 2×Laemmli sample buffer (Bio-Rad), 5% β-mercaptoethanol and 5% protease inhibitor (Millipore, USA). The cells were homogenized with a Precellys 24 (Bertin Corp., USA) under the following conditions: three cycles of 6,000 rpm for 40 sec and 2 min of cooling on ice. The samples were then incubated at 100°C for 5 min and cooled on ice for 5 min, and the supernatants were obtained by centrifugation at 13,000 g for 10 min. To confirm the expression of the FLAG-tagged HSbZIP1, the samples were electrophoresed using SDS polyacrylamide gel electrophoresis (PAGE) with a 4-15% gradient. The Trans-Blot Turbo system (Bio-Rad) was used to transfer the separated proteins to a polyvinylidene difluoride (PVDF) membrane. The membrane was blocked with 5% skim milk and 0.01% Tween 20 dissolved in phosphate-buffered saline (PBS) for 1 h, and then incubated for 1 h with keyhole limpet hemocyanin (KLH) anti-FLAG-tagged antibody (1:1000; Agrisera, Sweden). The membrane was then washed with skim milk solution and incubated for 1 h with horseradish peroxidase (HRP)-conjugated anti-mouse IgG (H&L) secondary antibody (1:1000; Agrisera). Protein bands were detected with Luminol/enhancer and Peroxide solution and the ChemiDoc system (Bio-Rad).

### Heterotrophic Culture Conditions

BG11 medium with glucose was used for heterotrophic cultivation. The medium consisted of 1.5 g/l NaNO_3_, 40 mg/l K_2_HPO_4_, 75 mg/l MgSO_4_·7H_2_O, 36 mg/l CaCl_2_·2H_2_O, 6 mg/l citric acid·H_2_O, 6 mg/l ferric ammonium citrate, 1 mg/l Na2EDTA, 20 mg/l Na2CO3, 1 ml BG11 trace metal solution (2.86 g/l H_3_BO_3_, 1.81 g/l MnCl_2_·4H_2_O, 0.22 g/l ZnSO_4_·7H_2_O, 0.39 g/l Na_2_MoO_4_·2H_2_O, 0.079 g/l CuSO_4_·5H_2_O, 49.4 mg/l Co(NO_3_)2·6H_2_O), and 10 g/l glucose. *Chlorella* sp. HS2 was inoculated at an optical density at 680 nm (OD_680_) of 0.3, which was estimated by a UV/Vis spectrophotometer (UV-1800; Shimadzu, Japan). The cells were cultivated in 500-ml Erlenmeyer baffled flasks with a 200 mL working volume at 37°C in the dark with continuous shaking at 120 rpm.

### Growth and Nutrient Analysis of the *HsbZIP1* Transformants

Cell growth was determined by the cell density and dry cell weight (DCW). Cell density was measured using a Cellometer Auto X4 Cell Counter (Nexcelom Bioscience, USA). The DCW was determined by filtering the cells through a GF/C filter paper (47 mm; Whatman, UK), washing the filters with deionized water, drying them overnight at 105°C, and weighing them on a fine scale.

The concentration of nitrate (NO_3_^-^) in the medium was determined by an ion chromatograph (881 Compact IC Pro; Switzerland) with a Metrosep A Supp5 150 column for anions. The concentration of glucose was determined by a high-performance liquid chromatograph (Dionex Ultimate 3000; ThermoFisher Scientific) with an Aminex HPX-87H column.

### Fatty Acid Methyl Ester Analysis

A chloroform-methanol mixture (2:1, v/v) was added to 10 mg of lyophilized cells, and the sample was mixed for 10 min for lipid extraction. Heptadecanoic acid (C17:0, 0.5 mg) was added as an internal standard. For transesterification, 300 μl of sulfuric acid and 1 mL of methanol were added and the mixture was incubated at 100°C for 20 min. The sample was then cooled, mixed with 1 ml of deionized water and centrifuged at 4,000 g for 5 min. The organic phase (lower layer) was obtained and filtered with a 0.20 μm RC-membrane syringe filter (Sartorius Stedim Biotech, Germany). The fatty acid methyl esters (FAMEs) were analyzed by a gas chromatograph (GC) (HP 6890; Agilent, USA) that had a flame ionization detector (FID) and an HP-INNOWax polyethylene glycol column (HP 19091 N-213; Agilent Technologies, Germany). The oven temperature of the GC was increased from 50 to 250°C at 15°C per min. The FAME composition and content were determined based on a 37-component mix of FAME standards (F.A.M.E. MIX C8-C24; Supelco, Sigma-Aldrich).

### Identification of *HsbZIP1* Targets and qRT-PCR Analysis of RNA Expression 

To identify *HsbZIP1* target genes, we used the four transcription factor binding sequences (TFBS) of *Chlorella* bZIP TF (TFmatrixID_0181, TFmatrixID_0190, TFmatrixID_0192, and TFmatrixID_0194) in the “PlantPAN 2.0” database (http://plantpan2.itps.ncku.edu.tw/index.html). The sequence patterns were predicted with RSAT ‘matrix-scan’ [26]. We obtained 2,265 predicted promoter sequences located within 600 bp upstream of the start codons of individual genes. Among these, we identified 1014 genes containing putative bZIP TFBS in their promoters. These 1014 genes were classified by the gene ontology (GO) term enrichment analysis, which was performed using the Blast2GO software (https://www.blast2go.com/) with Fisher’s exact test and a *p*-value < 0.05. For further assessment, we selected genes involved in fatty acid synthesis.

To confirm the expression levels of *HSbZIP1* and the predicted HSbZIP1 target genes, qRT-PCR was carried out. Total RNA was extracted using an RNeasy Plant Mini Kit (Qiagen, USA), and cDNA was synthesized using Superscript III Reverse Transcriptase (Invitrogen) and an oligo (dT)_20_ primer (Invitrogen). qRT-PCR was conducted with the CFX96 Real-Time system (Bio-Rad). The utilized primers are summarized in Table S1. The 18S rDNA was detected as a loading control for normalization. Each reaction solution consisted of 2 μl of cDNA, 0.5 μl of each primer (final concentration of each, 10 μM), 7 μl of deionized water and 10 μl of Universal SYBR Green Supermix (Bio-Rad). qRT-PCR was performed with the following steps: 95°C for 2 min; 40 cycles of denaturation at 95°C for 10 sec, annealing at 60°C for 10 sec, and extension at 72°C for 20 sec followed by denaturation at 95°C for 10 sec and a final melting step at 65–95°C. The gene expression level was analyzed by the 2^-ΔΔCt^ method, and statistical significance was assessed by the Student’s t test.

## Results

### Identification of bZIP Transcription Factors in *Chlorella* sp. HS2 and Screening of HSbZIP1 Transformants

In previous studies, the bZIP TF was predicted to be a key TF for fatty acid synthesis in *Nannochloropsis* [27], and our group showed that overexpression of the bZIP TF homolog, NsbZIP1, increased lipid production by regulating lipid synthesis genes in *N. salina* [10]. In the present study, we set out to identify and overexpress a homolog of this *Nannochloropsis* bZIP TF in *Chlorella* sp. HS2.

**Fig. 1:**
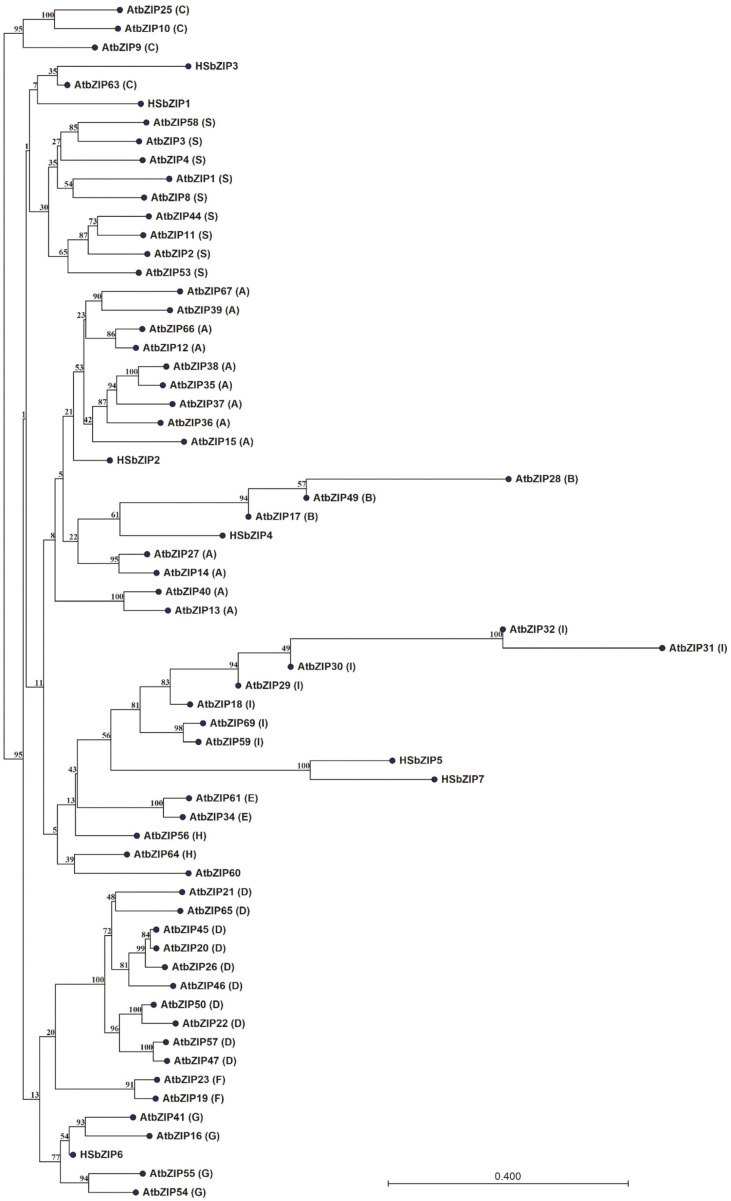
Phylogenetic tree of *Chlorella* sp. HS2 and *Arabidopsis* bZIP TFs. The phylogenetic tree of the aligned sequences was constructed with the maximum likelihood method using neighbor-joining, as applied with CLCbio Main Workbench. The HSbZIPs used in this study are listed in Table S2 and the *Arabidopsis* bZIPs were obtained from Jakoby *et al*.(2002) [29].

First, we found 13 bZIP TFs of *Chlorella variabilis* NC64A in “PlantTFDB” (http://planttfdb.cbi.pku.edu.cn/). Then, we identified seven bZIP TFs in *Chlorella* sp. HS2 by BLAST homology analysis based on the 13 bZIP TFs of *Chlorella variabilis* NC64A. We named them HSbZIP1 through HSbZIP7 (Table S2) and confirmed their bZIP domains using the Pfam database (https://pfam.xfam.org/) (Fig. S1). To assess the possible functions of the HSbZIP TFs, we performed phylogenetic analysis using *Arabidopsis* bZIP TFs (AtbZIP TFs) (Fig. 1) [28, 29]. The AtbZIP TFs are subdivided into 10 groups based on their domains and functions. Among them, C-type bZIPs regulate carbohydrate and lipid metabolism under stress conditions [30]. Indeed, NsbZIP1 TF, which is the C-type bZIP, contributed to increased lipid production in *N. salina* under stress conditions [10]. Using our AtbZIP TF-based phylogenetic analysis, we grouped the seven HSbZIP TFs (Fig. 1 and Table S2). Notably, HSbZIP1 and HSbZIP3 were associated with the C-type bZIP TFs of *Arabidopsis thaliana*. Finally, we selected HSbZIP1 due to the highest homology to NsbZIP1 of *N. salina* for further study [10].

**Fig. 2:**
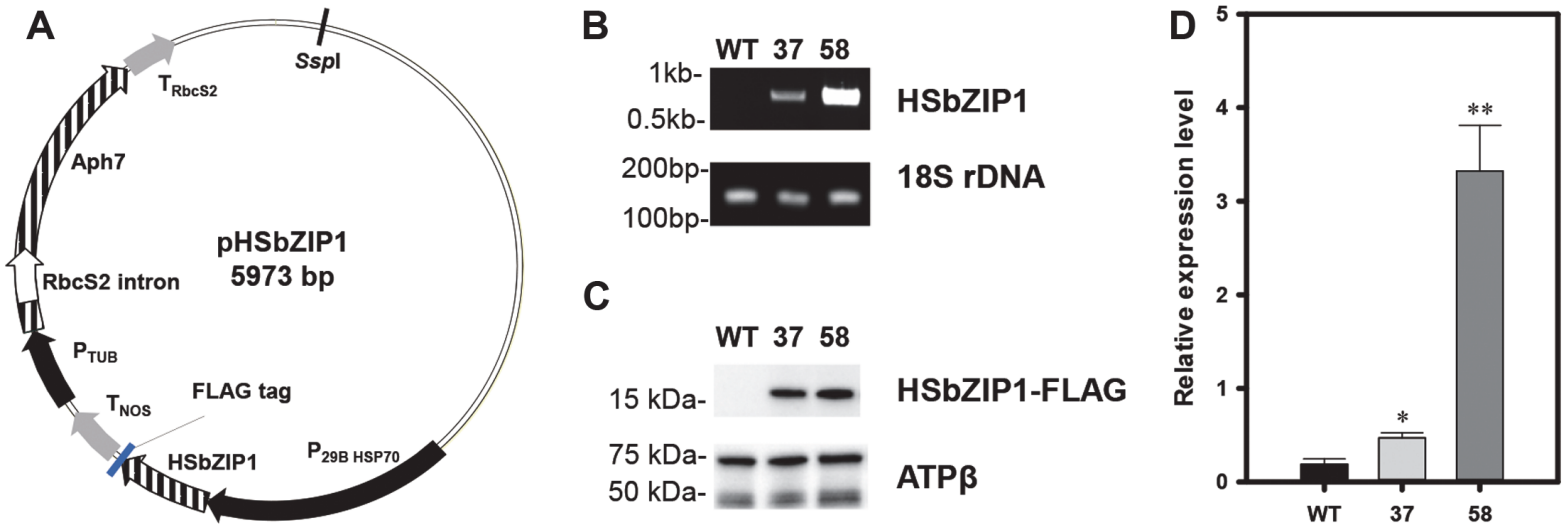
The pHSbZIP1 transformation vector and molecular identification of the HSbZIP1 transformants. (**A**) Schematic map of the pHSbZIP1 plasmid. (**B**) Detection of transgenes in the HSbZIP1 transformants and WT. The expected PCR product sizes of HSbZIP1 and the 18s rDNA were 754 and 139 bp, respectively. (**C**) Western blot analysis of FLAG-tagged HSbZIP1 in transformants. The expected size of FLAG-tagged HSbZIP1 was 14 kDa, but ran around 15 kDa. The β-subunit of ATP synthase (ATP β, experimental control) was used as a loading control. The expected sizes were 72.6 kDa (F-type H-ATPase β subunit) and 53.13 kDa (CF1 β subunit of ATP synthase). (**D**) The mRNA expressions levels of HSbZIP1. The expression levels were determined by qRT-PCR and normalized to the 18s rDNA. The data points represent the average of the samples, and the error bars indicate the standard error (*n* = 3). Significant differences against WT for the same condition and time point were determined by the Student’s *t*-test and are indicated by asterisks (*p < 0.05, **p < 0.01, ***p < 0.001).

To overexpress HSbZIP1 in *Chlorella* sp. HS2, we constructed pHSbZIP1, which contained the FLAG-tagged coding sequence and the *Aph7* gene as a hygromycin-selectable resistance marker (Fig. 2A). After transformation, we selected hygromycin-resistant colonies. We then conducted gDNA PCR to confirm the presence of the transgene and western blot analysis to identify the protein expression of the transgene by detecting the FLAG tag attached to the C-terminus of HSbZIP1 (Fig. S2). Ten transformants showing a specific PCR and a protein band were screened for growth and FAME production under heterotrophic conditions (Table S3). Basically, all transformants showed better growth and lipid production than WT. Particularly, as HSbZIP1 37 and 58 showed significantly increased FAME contents, we selected the two transformants for further analyses (Figs. 2B and 2C). Additionally, we investigated the mRNA expression levels of *HsbZIP1* in WT and the HSbZIP1 transformants, and found that the transformants showed higher expression levels of *HsbZIP1* compared to WT (Fig. 2D). Furthermore, the transcription levels of *HsbZIP1* were greater in HSbZIP1 58 than in HSbZIP1 37, suggesting that HSbZIP1 58 strain may show the better performance of lipid production than HSbZIP1 37.

### Effects of HSbZIP1 Overexpression on Biomass and FAME Yield Under Heterotrophic Cultivation

**Fig. 3:**
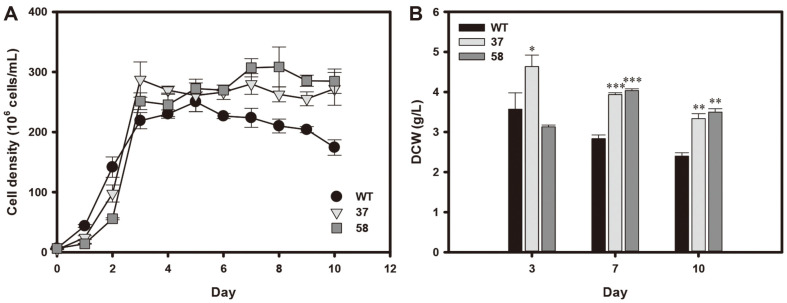
Growth analyses of the HSbZIP1 transformants under heterotrophic conditions. (**A**) Growth curve based on cell density. (**B**) DCW on days 3, 7 and 10. The data points represent the average of the samples, and the error bars indicate the standard deviation (*n* = 3). Significant differences against WT grown under the same condition were determined by Student’s *t*-test and are indicated by asterisks (*p < 0.05, **p < 0.01, ***p < 0.001).

We cultivated the HSbZIP1 transformants and WT in BG11 medium with 10 g/l of glucose in the absence of light for heterotrophic conditions. The HSbZIP1 transformants and WT reached stationary phase at day 3 according to cell density analysis (Fig. 3A). After day 5, the cell density decreased in WT but was maintained in the HsbZIP1 transformants during stationary phase. Likewise, the transformants showed higher DCW than WT on days 7 and 10 (Fig. 3B). The DCW of HSbZIP1 37 and 58 were 39% and 42% higher, respectively, than that of the WT on day 7 (Fig. 3B). Although the DCW of the WT and HSbZIP1 transformants decreased slightly on day 10 compared to day 7, the transformants still had a higher DCW than WT.

**Fig. 4:**
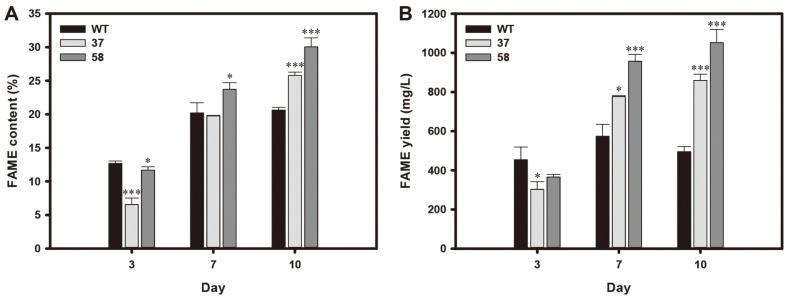
FAME analyses of the HSbZIP1 transformants under the heterotrophic conditions. FAME content (**A**) and FAME yield (**B**) were obtained on days 3, 7 and 10. The data points represent the average of the samples, and the error bars indicate the standard deviation (*n* = 3). Significant differences against WT grown under the same condition were determined by Student’s *t*-test and are indicated by asterisks (*p < 0.05, **p < 0.01, ***p < 0.001).

We then performed FAME analysis (Fig. 4). The FAME contents of the HSbZIP1 transformants were lower than those of WT on day 3 (Fig. 4A). However, while the FAME contents of WT were nearly identical on days 7 and 10, the two transformants continued to increase the FAME contents during the stationary phase. As a result, the FAME contents of HSbZIP1 37 and 58 were 26% and 46% higher, respectively, than that of WT on day 10 (Fig. 4A). Given their increased biomasses and FAME contents, the FAME yields of the transformants were significantly enhanced at the late stationary phase (Fig. 4B). The FAME yields of HSbZIP1 37 and 58 were 776 mg/l and 957 mg/l, respectively, on day 7, and were 35% and 67% higher, respectively, than that of WT at 574 mg/l. On the last day of cultivation (day 10), the FAME yields of HSbZIP1 37 and 58 were 859 and 1,052 mg/l, respectively, and were 74%and 113% higher, respectively, than that of WT at 495 mg/l (Fig. 4B).

We also investigated FAME compositions of the HSbZIP1 transformants (Table 1). The major fatty acids of *Chlorella* Sp. HS2 were palmitic acid (C16:0), oleic acid (C18:1), and linoleic acid (C18:2). As HSbZIP1 37 did not produce enough lipids on day 3, fatty acid compositions were different from other strains. However, the fatty acid compositions of WT and the two transformants were similar on days 7 and 10 where lipids were accumulated. Although the HSbZIP1 transformants showed slight decreases in C16:0 and C18:0 contents and an increase in C18:1 content relative to WT, there were no significant differences in FAME compositions of WT and the HSbZIP1 transformants. To confirm whether the HSbZIP1 TF affected biodiesel quality, we calculated the cetane number (CN), iodine value (IV) and degree of unsaturation (DU) (Table 1). A good biodiesel generally has a low IV and DU and a high CN [31]. On day 3, where lipids were not accumulated sufficiently, biodiesel qualities of the HSbZIP1 transformants were not considering the IV, DU, and CN values. On day 7 and 10, where lipids were accumulated, the CN, IV and DU of the HSbZIP1 37 were similar to those of WT. Noteworthily, HSbZIP1 58 showed a higher CN and a lower IV and DU compared to WT, indicating that it could generate better FAMEs for biodiesel production.

### Identification and Molecular Analysis of HSbZIP1-Regulated Candidate Genes Involved in Lipid Synthesis.

To investigate the mechanism underlying increased lipid production of the HSbZIP1 transformants, we performed a qRT-PCR of possible HSbZIP1-regulated genes. We screened the promoter regions of *Chlorella* sp. HS2 through TFBS analysis, and identified 1014 candidate genes that were predicted to be regulated by HSbZIP1. Among them, we selected for further analysis three genes involved in fatty acid synthesis: acetyl-CoA carboxylase 1 (ACC1), 3-ketoacyl-CoA synthase 4 and 11 (KCS4 and KCS11) (Table 2 and Fig. S3).

**Fig. 5:**
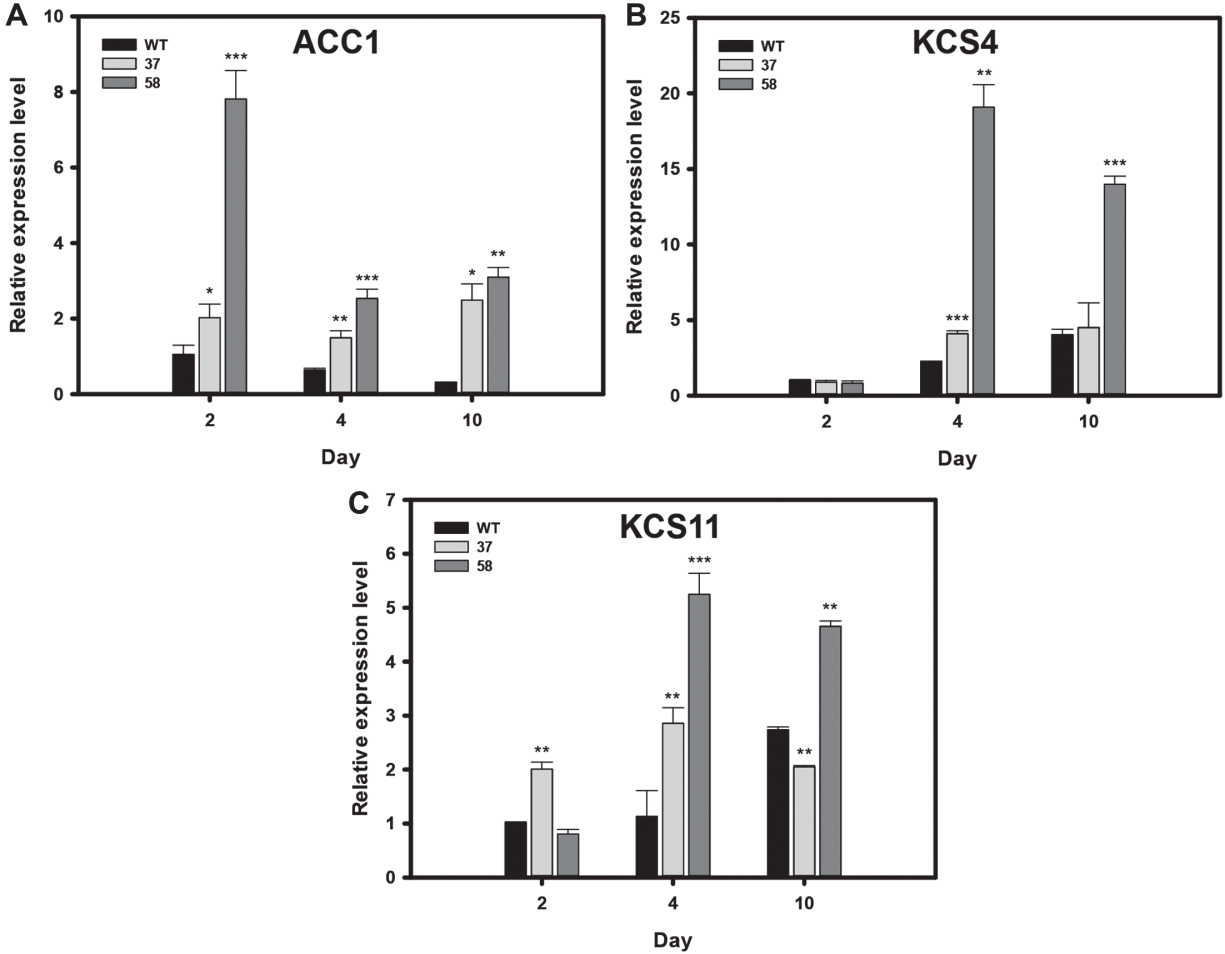
The expression profiles of HSbZIP1-regulated genes involved in lipid synthesis. The mRNA expression levels of *ACC1* (**A**), *KCS4* (**B**), and *KCS11* (**C**) were measured at days 2, 4 and 10 of growth under heterotrophic condition. The expression levels of the tested genes were determined by qRT-PCR and normalized to that of the 18s rDNA. Table 2 provides the full names of the studied genes. The data points represent the average of the samples, and the error bars indicate the standard error (*n* = 3). Significant differences against WT for the same condition and time point were determined by the Student’s *t*-test and are indicated by asterisks (*p < 0.05, **p < 0.01, ***p < 0.001).

We examined the mRNA expression levels of the selected genes on days 2, 4, and 10 under heterotrophic conditions. The fatty acid synthesis-related genes, ACC1, KCS4 and KCS11, exhibited higher expression levels in the HSbZIP1 transformants than in the WT during the cultivation (Fig. 5). In particular, HSbZIP1 58 showed significantly increased expression levels of these genes. This seems to explain the higher FAME contents and yields of HSbZIP1 58 compared to HSbZIP1 37 and WT (Fig. 4). Below, we will further discuss our interpretation of these results, together with the phenotypes of the HSbZIP1 transformants.

## Discussion

*Chlorella* spp. have been used in a variety of fields. For example, *Chlorella* has a positive effect on the immune system, and its powder is widespread in the health food market [32]. It is also widely used as a feed supplement for agriculturally important animals, due to its high protein content and antioxidant activity [33]. Given the advantages of *Chlorella* spp., many researchers have sought to engineer *Chlorella* as a host for the expression of heterologous proteins, including human growth hormones and various anti-cancer and antibacterial pharmaceutical products [34]. Furthermore, since *Chlorella* spp. can grow well under any culture condition (photoautotrophic, mixotrophic, and heterotrophic) with a high lipid content (14 to 63%) [35], various efforts have been made to use *Chlorella* spp. for biofuel production [13, 36].

In the present study, we performed the first genetic engineering of *Chlorella* sp. HS2 aimed at improving its potential for biofuel production. *Chlorella* sp. HS2 is considered a promising algal strain for biofuel production due to its strong growth, high biomass production and wide-ranging stress tolerance [22]. When compared to other *Chlorella* spp. (*e.g.*, *Chlorella vulgaris* UTEX-265, *Chlorella* sp. CCAP 211/53 and *Chlorella sorokiniana* HS1), *Chlorella* sp. HS2 has shown better performance for lipid production under autotrophic, mixotrophic and heterotrophic cultivation modes [22, 37]. Furthermore, cultivation systems and harvesting methods for *Chlorella* sp. HS2 have been developed to make the bioprocess for biofuel production economically feasible [37-40]. To improve the performance of this strain for lipid production, we herein applied transcription factor engineering to *Chlorella* sp. HS2.

We previously overexpressed the endogenous bZIP TF, NsbZIP1, in *N. salina*, and successfully increased biomass and lipid production [10]. Comparison to the *Arabidopsis* bZIP TFs indicates that NsbZIP1 can be classified as a C-type bZIP TF which is related to carbohydrate and lipid metabolism under stress conditions [30]. In the present study, we identified bZIP TFs in *Chlorella* sp. HS2, and selected HSbZIP1 which is a C-type bZIP with high homology to NsbZIP1 (Table S2). We overexpressed HSbZIP1 in *Chlorella* sp. HS2 with the goal of increasing lipid production. After genomic DNA PCR and western blotting analyses, we selected ten transformants expressing the transgenic HSbZIP1 and conducted phenotype-screening analysis of lipid production ability under heterotrophic conditions. Since the transformants were generated by random integration of the linearized HSbZIP1 expression vector, the expression of HSbZIP1 TF could be influenced by the genomic environment, resulting in different phenotypes of the transformants [41]. Nevertheless, most of the transformants (except for HSbZIP1 55) showed increases of FAME contents and yields (Table S3), indicating that the overexpression of HSbZIP1 TF was obviously beneficial for lipid production. We then selected two transformants, HSbZIP1 37 and 58, exhibiting significantly increased FAME contents compared to WT, for further study.

 We cultivated the selected transformants under heterotrophic conditions and verified their increased growth and lipid production. WT cells showed a decrease in cell density after day 5, whereas the HSbZIP1 transformants maintained their cell density (Fig. 3A). The transformants also enhanced the FAME contents during the stationary phase, while the FAME content of WT was maintained (Fig. 4A). Consequently, FAME production was greater in the HSbZIP1 transformants than in WT at the late stationary phase (Fig. 4B).

To understand the mechanism behind the increased lipid production of the HSbZIP1 transformants, we identified ACC1, KCS4 and KCS11, which are putative HSbZIP1-regulated genes involved in fatty acid synthesis. ACC converts acetyl-CoA to malonyl-CoA, and subsequently KCS catalyzes condensation reaction of long chain acyl (n ≥ 16) with malonyl-CoA [42]. Since the major fatty acids of the *Chlorella* sp. HS2 are long-chain fatty acids such as palmitic acid (C16:0), oleic acid (C18:1), and linoleic acid (C18:2) (Table 1), ACC and KCS might play critical roles in lipid production in *Chlorella* sp. HS2. Indeed, the HSbZIP1 transformants increased expression levels of ACC1, KCS4 and KCS11, resulting in enhanced FAME contents and yields relative to WT (Figs. 4 and 5). Additionally, we observed that FAME compositions of the HSbZIP1 transformants were not significantly altered compared to WT (Table 1). This means that upregulation of ACC and KCS seems to positively affect total fatty acid contents regardless of fatty acid compositions in the HSbZIP1 transformants.

We also noticed that the degree of HSbZIP1 expression affected lipid production. The mRNA expression levels of HSbZIP1 was considerably enhanced in HSbZIP1 58 (Fig. 2D). This could be related to the higher transcription levels of ACC and KCS in HSbZIP1 58 (Fig. 5), resulting in strong phenotypes in terms of lipid production (Fig. 4). These results suggest that the overexpression of HSbZIP1 TF upregulates ACC and KCS, resulting in an increase in total fatty acid production.

Hu *et al*. (2014) reported that the bZIP TF would regulate genes encoding 3-ketoacyl-ACP synthase, long-chain acyl-CoA synthase and acyl-CoA-binding proteins in *Nannochloropsis* [27]. In the previous study, we demonstrated that the genes were upregulated by overexpression of the bZIP TF in *N. salina*, resulting in an increase in lipid production [10]. Although the HSbZIP1-regulated genes differed somewhat with respect to the NsbZIP1-regulated genes, HSbZIP1 seems to play a similar function to NsbZIP1, regulating the fatty acids synthesis pathway [10, 27].

In efforts to improve growth and lipid production of *Chlorella* sp. HS2, various cultivation methods have been reported [22,37-39,43]. Particularly, Yun *et al*. (2019) and Kim *et al*. (2019) reported optimized heterotrophic conditions for *Chlorella* sp. HS2 [22, 43]. Interestingly, they showed increased biomass production and altered fatty acid profiles using yeast extract or high concentration of glucose. Although it is not known how the characteristics of the HSbZIP1 transformants will change under the optimized heterotrophic conditions, it is expected that lipid production of the HSbZIP1 transformants could be further enhanced.

In summary, we herein report the first successful transcription factor engineering of *Chlorella* sp. HS2. Overexpression of the endogenous TF, HSbZIP1, enhanced the FAME yield in *Chlorella* sp. HS2 under heterotrophic cultivation, in association with changes in putative target genes involved in fatty acid synthesis. Although further studies for a mechanistic perspective on the increased biomass and lipid production of the HSbZIP1 transformants are needed, our present results suggest that transcription factor engineering with HSbZIP1 could be employed in *Chlorella* sp. HS2 to support biofuel production.

## Supplemental Materials



Supplementary data for this paper are available on-line only at http://jmb.or.kr.

## Figures and Tables

**Table 1:** Analyses of the fatty acid composition and the biodiesel quality of the HSbZIP1 transformants.

Strains	Day	Fatty acid composition (%)							CN1^a^	CN2^b^	IV^c^	DU^d^

		C16:0	C16:1	C18:0	C18:1	C18:2	C18:3	etc				
WT	Day 3	22.8 ± 0.2	1.7 ± 0.0	6.4 ± 0.4	27.9 ± 0.3	35.8 ± 0.1	n.d.	5.4 ± 0.1	54.0 ± 0.0	53.1 ± 0.1	87.6 ± 0.2	101.2 ± 0.2
37		25.9 ± 3.3	n.d.	n.d	n.d	32.8 ± 3.5	12.0 ± 10.4	29.3 ± 3.6.	51.8 ± 3.0	51.6 ± 2.6	88.3 ± 17.3	89.6 ± 11.2
58		24.0 ± 0.4	2.4 ± 0.2	3.0 ± 0.4	22.3 ± 0.5	41.7 ± 0.5	n.d.	6.6 ± 0.2	53.4 ± 0.1	51.6 ± 0.1	93.6 ± 0.3	108.0 ± 0.4
												
WT	Day 7	26.8 ± 0.6	1.6 ± 0.2	4.8 ± 0.2	20.3 ± 0.2	38.7 ± 0.4	1.2 ± 0.0	6.6 ± 0.6	53.7 ± 0.2	52.8 ± 0.3	89.4 ± 1.0	101.9 ± 1.1
37		22.9 ± 0.1	1.8 ± 0.1	3.0 ± 0.1	23.4 ± 0.2	40.7 ± 0.2	0.0 ± 0.0	8.1 ± 0.1	53.3 ± 0.1	51.6 ± 0.1	92.4 ± 0.5	106.6 ± 0.6
58		23.9 ± 0.4	2.2 ± 0.0	3.5 ± 0.1	25.8 ± 0.6	33.9 ± 0.2	0.2 ± 0.3	10.5 ± 0.6	54.9 ± 0.1	53.4 ± 0.1	83.5 ± 0.9	96.2 ± 0.8
												
WT	Day 10	26.5 ± 0.5	1.7 ± 0.1	3.9 ± 0.3	19.4 ± 0.3	40.8 ± 0.5	1.2 ± 0.0	6.4 ± 0.5	53.3 ± 0.2	52.1 ± 0.3	92.1 ± 1.3	105.1 ± 1.4
37		23.6 ± 0.1	2.0 ± 0.0	3.0 ± 0.1	20.9 ± 0.2	43.1 ± 0.1	0.0 ± 0.0	7.5 ± 0.1	53.0 ± 0.1	51.3 ± 0.1	94.4 ± 0.4	108.9 ± 0.5
58		23.8 ± 0.1	2.2 ± 0.1	3.3 ± 0.1	23.6 ± 0.5	36.7 ± 0.5	0.7 ± 0.0	9.7 ± 0.3	54.2 ± 0.1	52.96 ± 0.2	87.7 ± 0.6	100.5 ± 0.7

^a^The cetane number 1 (CN1) was calculated as follows: CN-1 = 61.1 + 0.088x_2_ + 0.133x_3_ + 0.152x_4_ − 0.101x_5_ − 0.039x_6_ − 0.243x_7_ − 0.395x_8_, where x_2_ to x_8_ indicated the weigh percentage of methyl esters as follows: C14:0, C16:0, C16:1, C18:0, C18:1, C18:2 and C18:3, respectively [44].

^b^The cetane number 2 (CN2) was calculated as follows; CN-2 = 62.2 + 0.017*L* + 0.074*M* + 0.115*P* + 0.177*S* − 0.103*O* − 0.279*LI* − 0.366*LL*, where *L*, *M*, *P*, *S*, *O*, *Li*, and *LL* indicated the weight percentages of the methyl esters as follows: C12:0, C14:0, C16:0, C18:0, C18:1, C18:2 and C18:3, respectively [45].

^c^The iodine value (IV) was determined according to the European standard method (EN 14214).

^d^The degree of unsaturation (DU) = 1 (monounsaturated Cn: 1, wt.%) + 2 (polyunsaturated Cn: 2, 3, wt.%) [46]. n.d., not detected.

**Table 2:** Lipid synthesis genes whose promoters contain bZIP TF binding sites.

Gene name^a^	Gene ID^b^	Abbreviation	GO Names (ID) list^b^
Acetyl-CoA carboxylase 1-like	HScell_00001718	ACC1	F: acetyl-CoA carboxylase activity; F: ATP binding; P: fatty acid biosynthetic process; F: ligase activity; F: metal ion binding
3-ketoacyl-CoA synthase 4-like	HScell_00002599	KCS4	F: catalytic activity; P: fatty acid biosynthetic process; P: metabolic process; C: membrane; F: transferase activity, transferring acyl groups other than amino-acyl groups
3-ketoacyl-CoA synthase 11	HScell_00003749	KCS11	F: catalytic activity; P: fatty acid biosynthetic process; P: metabolic process; C: membrane; F: transferase activity, transferring acyl groups other than amino-acyl groups

^a^Gene names and Gene ID refer to *Chlorella* sp. HS2 database (http://web.seeders.co.kr/hs2/index.php/chl/browse).

^b^GO enrichment test was done with the Blast2GO software, Fisher’s exact test and a *p*-value < 0.05.

^*^F, molecular function

^*^P, biological process
