# Supplementary material for: Enhancement of Lipid Production under Heterotrophic Conditions by Overexpression of an Endogenous bZIP Transcription Factor in Chlorella sp. HS2
Source: J Microbiol Biotechnol. 2020 Jul 29;30(10):1597–606. doi: 10.4014/jmb.2005.05048 (PMC9728203; doi:10.4014/jmb.2005.05048)
Supplement: Supplementary file 1 [file JMB-30-10-1597-supple.pdf]

J. Microbiol. Biotechnol.
https://doi.org/10.4014/jmb.2005.05048

Supplementary data

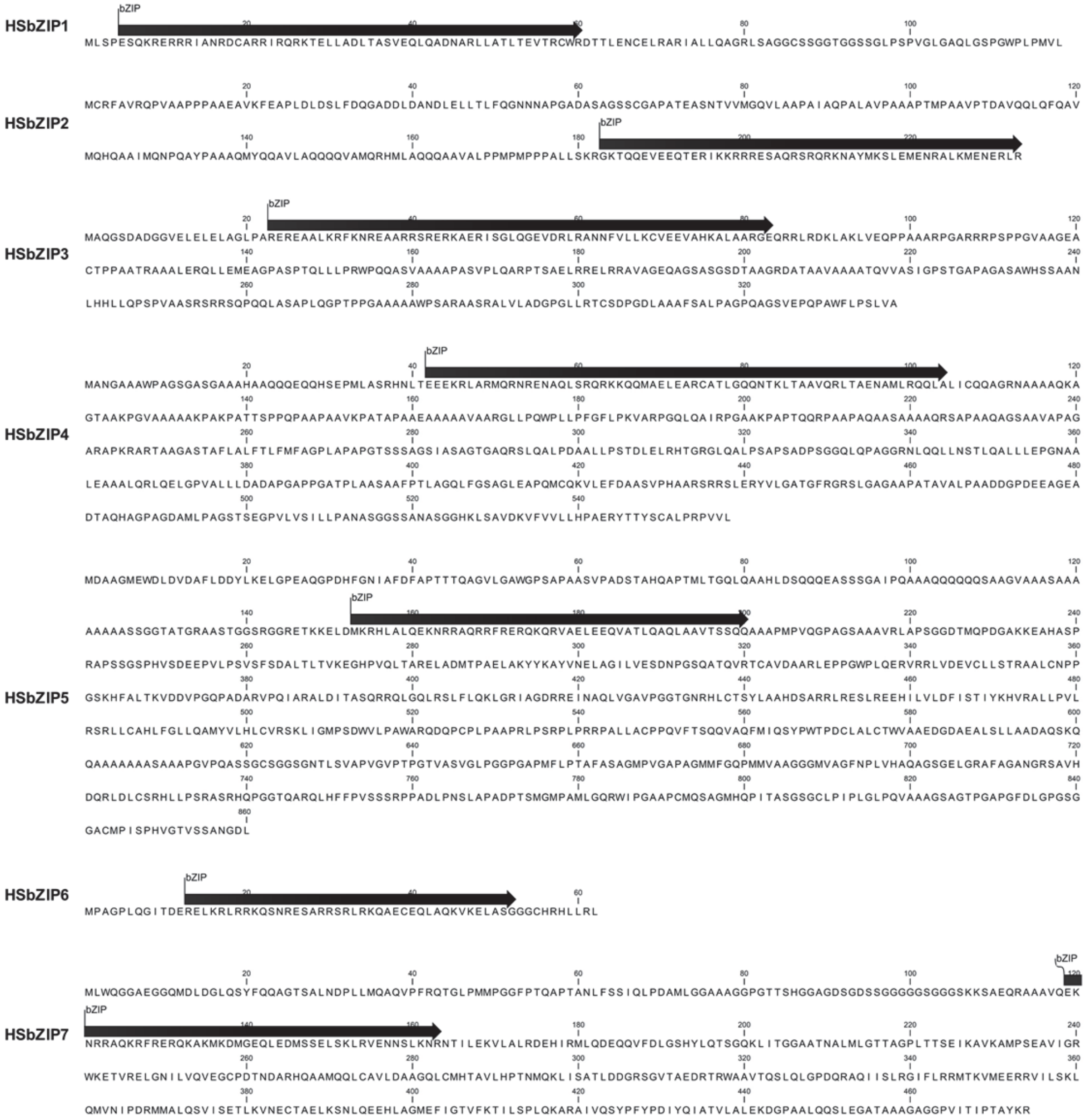

Fig. S1. Amino acid sequences of the bZIP transcription factor domain-containing proteins in *Chlorella* sp. HS2. bZIP domains were determined by searching the Pfam database (https://pfam.xfam.org/).

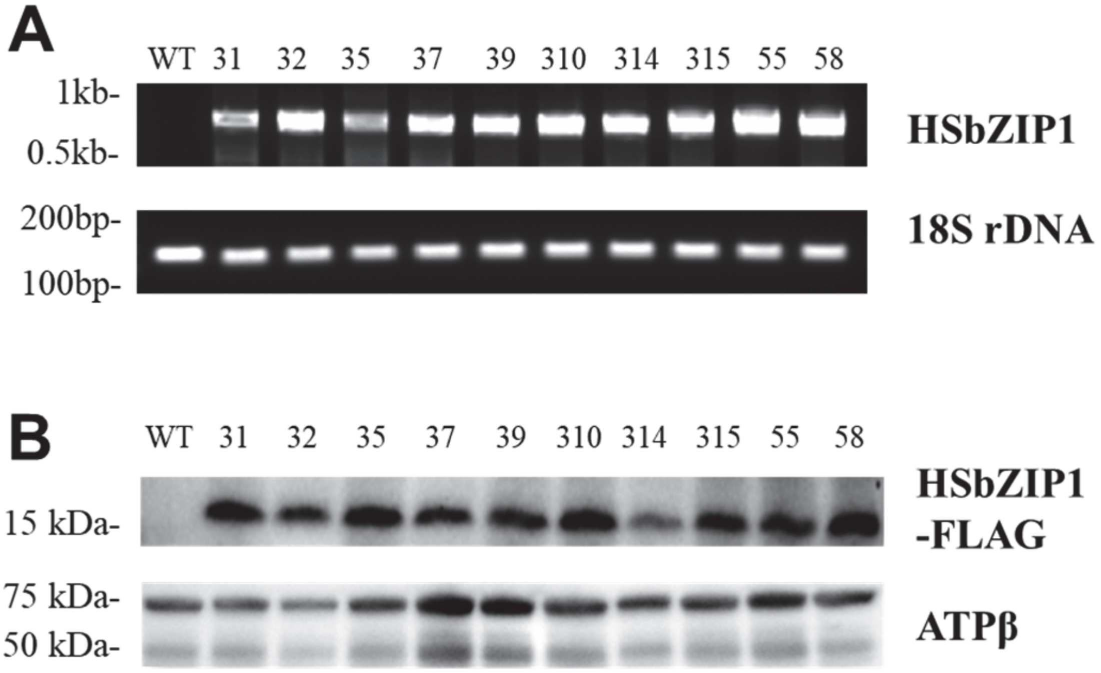

Fig. S2. Molecular identification of the HSbZIP1 transformants. (A) Detection of transgenes in HSbZIP1 transformants and WT. The expected PCR product sizes of HSbZIP1 and the 18s rDNA were 754 and 139 bp, respectively. (B) Western blot analysis of FLAG-tagged HSbZIP1 in transformants; it had an expected molecular weight of 14 kDa but ran around 15 kDa. The β-subunit of ATP synthase (ATP β, experimental control) was used as a loading control; its expected sizes were 72.6 kDa (F-type H-ATPase ß subunit) and 53.13 kDa (CF1 ß subunit of ATP synthase).

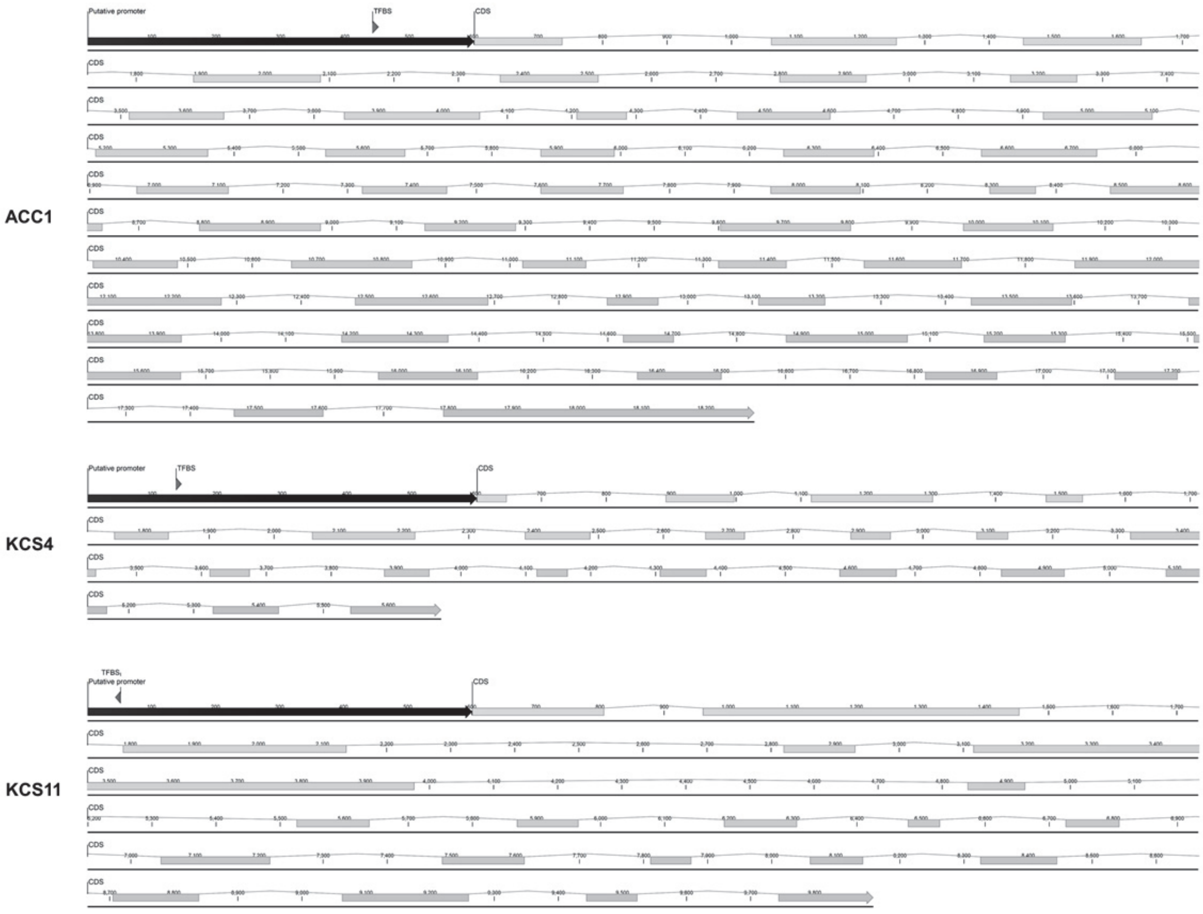

Fig. S3. Schematics of *Chlorella* sp. HS2 fatty acid synthesis-related genes containing bZIP TF binding sites in their promoters. TFBS, transcription factor binding sites; CDS, coding sequence.

Table S1. Primers used in this study.

Primer	Sequence (5'→3')	Purpose
HSbZIP1-fwd	CGCTTCTGTCACCTGCAACCATGCTCTCGCCCGAGAGCCA	Amplifying HSbZIP1 cDNA for Gibson assembly
HSbZIP1-rev	TTGAACGATCGGGGAAATTCGAGCTCTCACTTGTCGTCGTCGTCCTTGTAGTCGAGCACCATGGGCAATGGCC	
pHSbZIP1-fwd	TTGAACGATCGGGGAAATTCGAGCTCTCACTTGTCGTCGT	Amplifying pHSbZIP1 backbone for Gibson assembly
pHSbZIP1-rev	TGGCTCTCGGGCGAGAGCATGGTTGCAGGTGACAGAAGCG	
CPC1-fwd	ACGGAAGAAACTTGGCCGCCGACAAATT	Genomic DNA PCR for HSbZIP1
CPC1-rev	ATCTAGTAACATAGATGACACCGCGCGCGA	
18S-1-fwd	TGATTCCGGTAACGAACGAG	Genomic DNA PCR for 18S rDNA
18S-1-rev	CATCTAAGGGCATCACAGAC	
q18S_fwd	TGATTCCGGTAACGAACGAG	qRT-PCR for 18S rDNA
q18S_rev	CATCTAAGGGCATCACAGAC	
qp1_fwd	ACTCGCCCATCGACTTTGAG	qRT-PCR for ACC1
qp1_rev	AGAAGTAGCCCCACACCTCT	
qp2_fwd	CTGGTACCACGGTATCGAGC	qRT-PCR for KCS4
qp2_rev	GGTATGCGGTGTCGTCTGAG	
qp3_fwd	CAACTGCTCGCTGTTCAACC	qRT-PCR for KCS11
qp3_rev	CCAGGCCGATGGAAATGACT	
qHSbZIP-fwd	GGCTGATCTAACAGCAAGCG	qRT-PCR for HSbZIP1
qHSbZIP-rev	GCAGTTCTCCAGGGTGGTAT	

Table S2. Features of the HSbZIP transcription factors.

Given name	Type[a]	Peptide length (aa)	Reference gene ID[b]	GenBank accession number[c]
HSbZIP1	C	119	Hscell_00006746	MN593349
HSbZIP2	A	233	Hscell_00002364	MN593350
HSbZIP3	C	339	Hscell_00002410	MN593351
HSbZIP4	B	559	Hscell_00003265	MN593352
HSbZIP5	I	860	Hscell_00004284	MN593353
HSbZIP6	G	63	Hscell_00005993	MN593354
HSbZIP7	I	474	Hscell_00006563	MN593355

[a]The type of HSbZIP was assigned based on the phylogenetic analysis presented in Fig. 1.

[b]Described gene ID was found in *Chlorella* HS2 by homology search using the *Chlorella* HS2 genome portal:

(http://web.seeders.co.kr/hs2/index.php/chl/browse)

[c]GenBank accession numbers have been assigned for the CDS.

Table S3. Phenotype screening of the HSbZIP1 transformants under heterotrophic condition.

Strain	DCW (g/L)	FAME content (%)	FAME yield (mg/L)
WT	2.8	15.1	422.5
31	4.0	25.5	1021.2
32	3.6	24.4	877.3
35	3.9	28.0	1093.6
37	3.4	29.1	990.8
39	4.0	24.1	962.0
310	4.0	17.3	690.0
314	4.1	24.6	1010.2
315	4.8	25.2	1196.5
55	2.9	14.7	425.1
58	3.6	32.0	1150.2

Cells were cultivated under heterotrophic conditions for 10 days. The data were obtained at day 10.
